# PET/CT background noise and its effect on speech recognition

**DOI:** 10.1038/s41598-021-01686-5

**Published:** 2021-11-11

**Authors:** Iva Speck, Valentin Rottmayer, Konstantin Wiebe, Antje Aschendorff, Johannes Thurow, Lars Frings, Philipp T. Meyer, Thomas Wesarg, Susan Arndt

**Affiliations:** 1 Department of Otorhinolaryngology, Head and Neck Surgery, Medical Center—University of Freiburg, Faculty of Medicine, University of Freiburg, Killianstraße 5, 79106 Freiburg, Germany; 2 Department of Nuclear Medicine, Medical Center—University of Freiburg, Faculty of Medicine, University of Freiburg, Hugstetter Straße 55, 79106 Freiburg, Germany

**Keywords:** Preclinical research, Auditory system

## Abstract

Positron emission tomography (PET) has been successfully used to investigate central nervous processes, including the central auditory pathway. Unlike early water-cooled PET-scanners, novel PET/CT scanners employ air cooling and include a CT system, both of which result in higher background noise levels. In the present study, we describe the background noise generated by two state-of-the-art air-cooled PET/CT scanners. We measured speech recognition in background noise: recorded PET noise and a speech-shaped noise applied in clinical routine to subjects with normal hearing. Background noise produced by air-cooled PET/CT is considerable: 75.1 dB SPL (64.5 dB(A)) for the Philips Gemini TF64 and 76.9 dB SPL (68.4 dB(A)) for the Philips Vereos PET/CT (Philips Healthcare, The Netherlands). Subjects with normal hearing exhibited better speech recognition in recorded PET background noise compared with clinically applied speech-shaped noise. Speech recognition in both background noises correlated significantly. Background noise generated by PET/CT scanners should be considered when PET is used for the investigation of the central auditory pathway. Speech in PET noise is better than in speech-shaped noise because of the minor masking effect of the background noise of the PET/CT.

## Introduction

Since the 1980s, non-invasive functional imaging modality positron emission tomography (PET) has been used to investigate physiological (e.g., cerebral blood flow), biochemical (e.g., metabolism), and molecular (e.g., receptor binding) processes in living humans. PET can be applied in combination with a variety of radiotracers, e.g., with oxygen-15 labeled water ([^15^O]water) quantitatively to map the regional cerebral blood flow or with fluorine-18 labeled fluorodeoxyglucose ([^18^F]FDG) quantitatively to map regional glucose metabolism.

PET has been successfully used for many years to investigate central nervous processes, including the central auditory pathway^1–6^. Use of the neuroimaging technique PET in the auditory sciences has many advantages as summarized by Talvage et al.^7^: (1) it is non-confining and therefore suitable for subjects with traits of claustrophobia, (2) it is non-magnetic and therefore compatible with cochlear implants and hearing aids, and (3) it is quiet and therefore does not interfere with auditory stimuli.

Cochlear implants are implantable neuro-prothesis that can rehabilitate hearing in subjects with hearing impairments or in subjects with residual hearing. The cochlear implant consists of an internal part (electrode array, receiver, and magnet) and an external part (speech processor, microphone, and transmitter)^8^. Dislocation of the internal cochlear implant magnet can occur when functional magnetic resonance imaging (fMRI) is used. In contrast, PET entails no risk of dislocation. Nevertheless, even with the new generation of MRI-compatible cochlear implant magnets, the magnet of an implant can cause an artifact on MRI scans^9–11^.

PET is described as a comparatively quiet imaging technique that interferes little or not at all with acoustic stimuli other than fMRI with an ambient noise of up to 130 dB^9^. However, compared with early stand-alone water-cooled PET-scanners, novel clinical PET/CT scanners are air-cooled and include a CT system, both of which result in higher background noise levels.

The primary aim of the present study has therefore been to describe the background noise generated by two representative state-of-the-art air-cooled PET/CT scanners: the Gemini TF64 and Vereos PET/CT (Philips Healthcare, The Netherlands). Auditory stimulation studies in PET/CT scanners that are air-cooled have to take background noise into consideration, as it interferes with the presented auditory target signals. A description of the noise produced by novel PET/CT is therefore necessary for future studies of the auditory system.

We have also investigated the influence of the background noise produced by air-cooled PET/CT scanners on speech recognition in young adults with normal hearing.

Numerous studies have applied speech stimuli during PET scanning to investigate responses of the central auditory pathway^2,12–18^. To our knowledge, none of these publications mention possible effects of PET background noise on these responses. For example, Berding et al.^12^ correlated speech recognition measured in silence before PET scanning with voxel analysis of PET scans acquired for acoustic presentation of speech stimuli during scanning. This approach has a potential bias: images of neural responses obtained for presentation of speech in PET noise might be only poorly correlated with speech recognition assessed in quiet. Similarly, Coez et al.^13^, for example, separated their study participants into good and poor performers based on speech recognition acquired in quiet instead of in PET background noise thereby causing a potential bias. During PET scanning, the study participants passively listened to voice and non-voice stimuli^13^.

We have therefore chosen to investigate speech recognition in two kinds of background noise: in background noise of the Vereos PET/CT scanner (Philips Healthcare, The Netherlands) and in speech-shaped noise applied for speech recognition assessment during clinical routine. We compare and correlate speech performance between both noises to estimate the magnitude of such potential bias. Our hypothesis is that the mechanical background noise of PET interferes differently and possibly to a less extent with speech recognition than does speech-shaped background noise.

## Methods and materials

The present study was conducted in accordance with the guidelines of the Declaration of Helsinki (Washington, World Medical Association, 2013), was approved by the ethics committee of the University of Freiburg (310/17), and was registered in the German clinical trials register (DRKS00015477). All included adults gave informed consent before inclusion in the present study.

### Subjects

Ten adults with normal hearing (5 male and 5 female) aged between 20 and 39 years (27.37 ± 5.47 years) participated in the present study (Table 1). Subjects were recruited via public notifications in the University Hospital Freiburg and the University Freiburg.

**Table 1:** Characteristics of included subjects.

Subject ID	Age at testing (years)	Sex	Air conduction PTA4 [dB HL]		Speech reception threshold in background noise of Oldenburg sentence test [dB SNR]	Speech reception threshold in PET noise [dB SNR]
			Right ear	Left ear		
NH1	26.1	Male	8.75	12.5	− 7.9	− 11.5
NH2	25.1	Female	6.25	2.5	− 7.0	− 10.7
NH3	39.3	Female	10	10	− 6.0	− 9.1
NH4	22.3	Female	6.25	6.25	− 5.5	− 8.8
NH5	26.1	Female	12.5	18.75	− 6.7	− 10.8
NH6	26.1	Male	3.75	3.75	− 5.3	− 8.9
NH7	20.7	Male	6.25	6.25	− 6.1	− 10.4
NH8	32.7	Female	10	10	− 5.2	− 9.6
NH9	24.6	Male	13.75	16.25	− 5.9	− 8.6
NH10	30.7	Male	20	30	− 4.6	− 6.9

**Figure 1:**
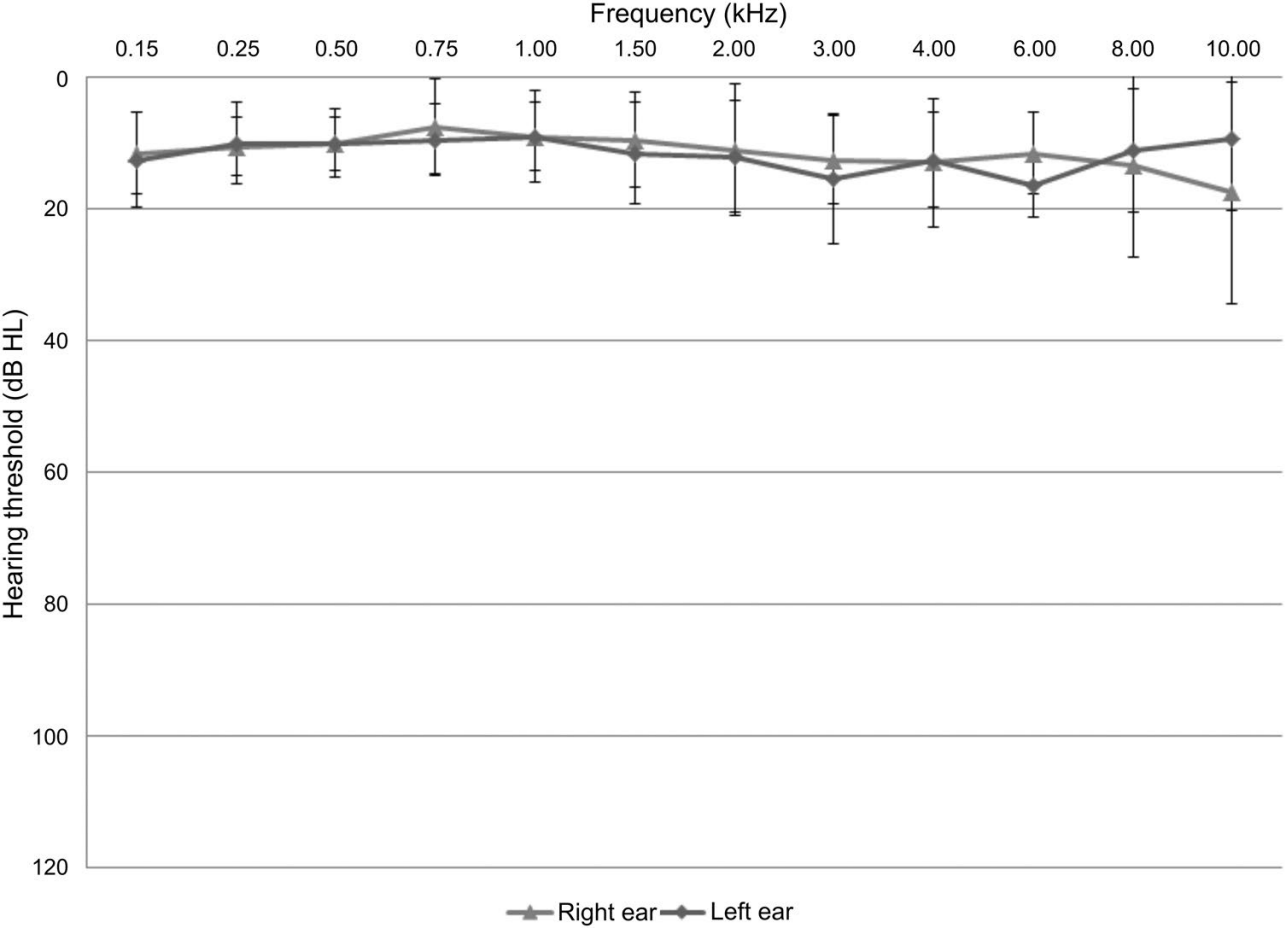
Mean hearing threshold levels ± standard deviations for subjects with normal hearing.

Normal hearing was judged by air-conduction hearing thresholds. A mean threshold for the frequencies 500, 1000, 2000, or 4000 Hz (PTA4) lower than or equal to 20 dB HL was required (Table 1; Fig. 1). Threshold measurements were carried out in a sound-isolated booth by means of a clinical audiometer AT1000 device (AURITEC Medizindiagnostische Systeme GmbH, Germany). We measured the bone-conduction and air-conduction threshold with sine tones between 250 Hz and 8 kHz. The frequency-dependent, subjectively just audible tone was specified as the hearing threshold.

#### PET noise

PET background noise generated by the air-cooled Gemini TF64 or Vereos PET/CT (Philips Healthcare, The Netherlands) was recorded in the scanner room (702 × 468 × 245 cm) with a Nor140 sound level meter (Norsonic Tippkemper, Germany). The Nor140 was positioned on a tripod at the height of the scanner table and was aimed directly at the head of the scanner opening. In addition, the background noise of the Vereos PET/CT (790 × 453 × 240 to 248 cm) was recorded with the head and torso simulator KEMAR (Knowles Electronic Manikin for Acoustic Research, GRAS Sound & Vibration A/S, Denmark) by using the in-built microphones. The KEMAR was placed on the table of the PET/CT scanner, with the head in the head rest, and was placed in the same position as the subjects undergoing PET scans. The background noise was recorded separately for both ear canals of the KEMAR with the ear canals open. We performed additional recordings with the ear canals blocked with either OHROPAX^®^ Soft or OHROPAX^®^ Silicon Aqua (OHROPAX GmbH, Germany) to measure the reduction of stimulus level achieved following the application of in-ear hearing protection. We recorded the background noise for each condition described above. Recordings with the KEMAR lasted for one minute and with the Nor140 lasted for 5 min. We repeated the measurement of background noise of the PET/CT Vereos Scanner at a separate date. The mean background noise level was computed for each frequency and in total from the averages of the two sound pressures related to the noise levels obtained during both background noise measurements. Additional repeated measurements over time were not available.

Of note, noise recordings were not performed during CT scanning (a source of considerable additional noise), i.e., all scans were carried out with the CT system on stand-by. We decided on the latter course of action because CT takes place only once, namely at the beginning of the PET/CT scan, for attenuation correction and lasts approximately 1 min. Thus, during the subsequent PET scan, i.e., the time at which any auditory stimulation of the subjects would occur, only the PET background noise recorded in this study would be present.

### Speech recognition

Speech reception thresholds in noise were assessed for sentences of the Oldenburg sentence test (OLSA)^19,20^. The sentences of the Oldenburg sentence test are composed of five words: name–verb–numeral–adjective–object. The sentences are combined at random from ten possible words per position. The resulting sentences are grammatically correct but semantically unpredictable. The participants were asked to repeat the sentences and guess words if unsure.

We measured speech reception thresholds in two different background noises: the background noise of the Oldenburg sentence test (OLnoise) and the background noise of the Vereos PET/CT (PET noise). OLnoise is speech-shaped noise and was created by overlaying the randomly shifted speech material of the Oldenburg sentence test thirty times (amplitude spectrum see HörTech gGmbH^21^). The long-term spectrum of the OLnoise corresponds to the long-term spectrum of the sentence corpus of the Oldenburg sentence test and the middle long-term spectrum of most languages (long-term average speech spectrum after Byrne et al.^22^). The PET background noise recorded with the Nor140 from the Vereos PET/CT was used as the second background noise: PET noise.

Before being tested, each participant underwent training (1) in the quiet, (2) in OLnoise, and (3) in PET noise. After training, we tested each subject in OLnoise and PET noise, with one randomly selected list of 30 OLSA sentences each. After the training session, the speech reception threshold for 50% correct word recognition was measured following the A1 procedure described by Brand and Kollmeier^23^ (p. 2804).

We presented speech and background noises from a loudspeaker Genelec 8030B (Genelec Oy, Finland) at a 1-m distance in front of the subject in a sound-proof booth. Speech reception thresholds were determined at a fixed noise level of 76.9 dB SPL and an initial speech level of 76.9 dB SPL. Presentation levels were calibrated at the microphones of the KEMAR placed at the location of the head of the subject but without the subject being present. The initial speech and noise levels were chosen according to the level of the PET noise (76.9 dB SPL) measured with the Nor140. The speech reception threshold was calculated as the difference between the speech and noise levels allowing 50% correct word-recognition.

## Results

**Figure 2:**
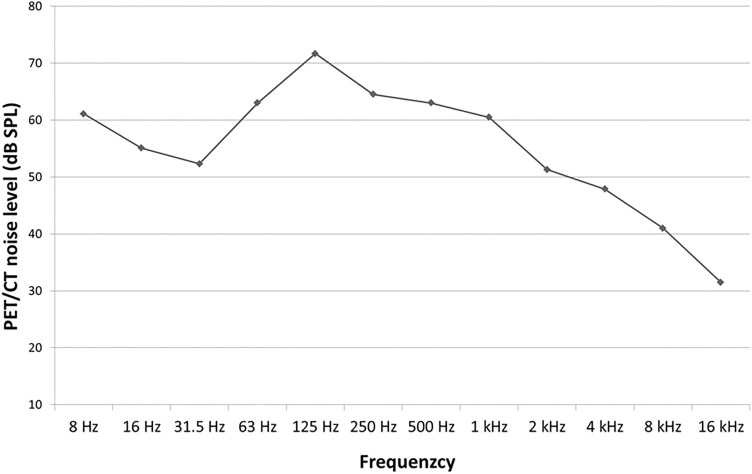
Amplitude spectrum of PET noise recorded from the Gemini TF64.

We recorded, in the scanner room of the Gemini TF64, a noise level of 75.1 dB SPL (64.5 dB(A)) with the Nor140 (amplitude spectrum in Fig. 2).

**Figure 3:**
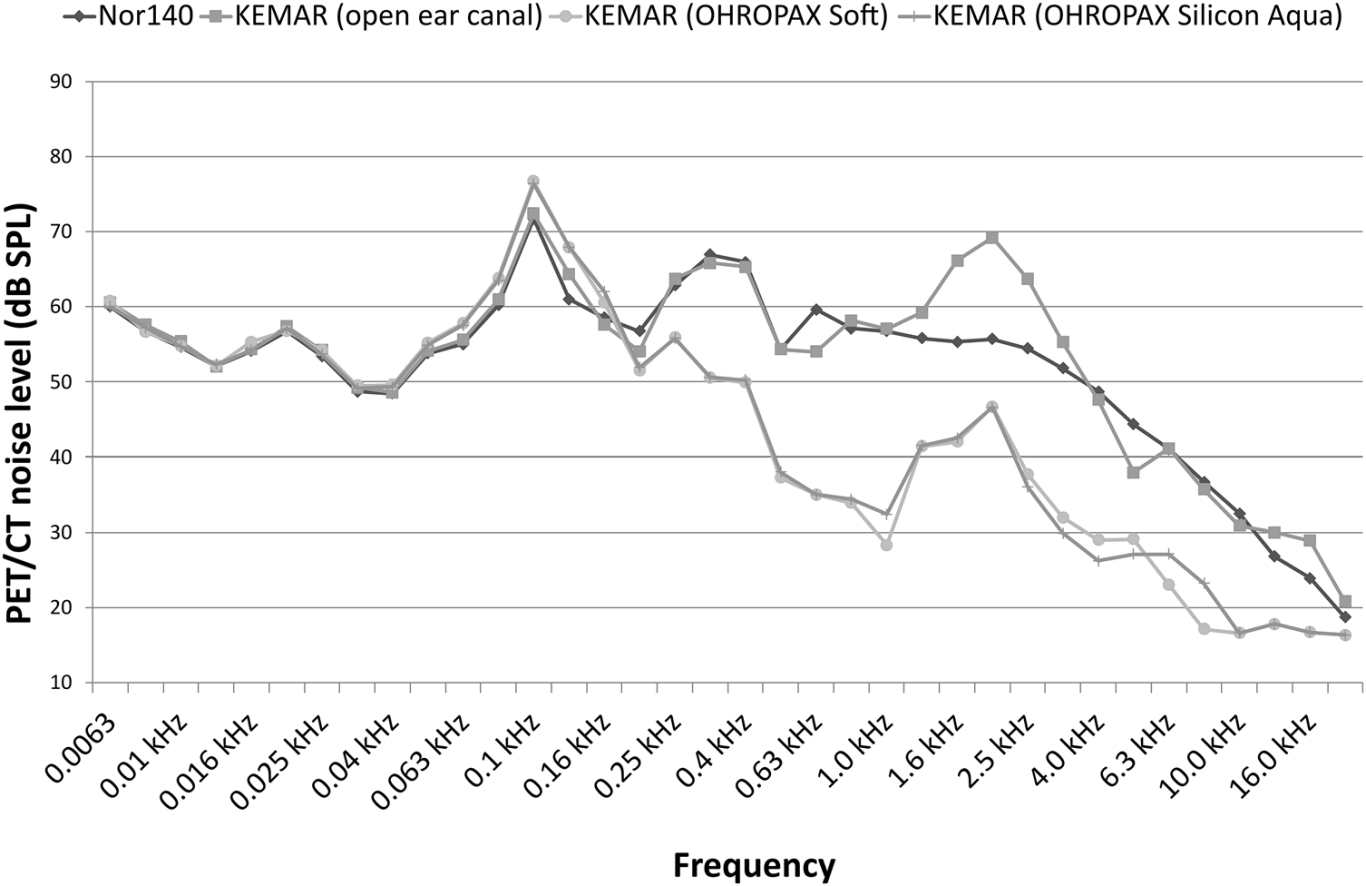
Amplitude spectrum of PET background noise recorded from the Vereos PET/CT.

The fully digital Vereos PET/CT produced a mean noise level of 76.41 dB SPL (67.24 dB(A)) when recorded with the Nor140. The mean PET background noise recorded with the KEMAR (ear canal open) for both ears separately was 79.10 dB SPL (74.29 dB(A)) in the left ear and 79.31 dB SPL (73.57 dB(A)) in the right ear. The mean amplitude spectrum averaged over both ears is displayed in Fig. 3 (KEMAR, ear canal open). The PET background noise is composed of mostly lower and medium frequencies (Fig. 3). For each noise, namely PET noise and OLnoise, temporal characteristics were assessed as the standard deviation of the root mean square (RMS) value and the mean and standard deviation of the CREST factor, the ratio between the absolute maximum amplitude and the RMS, obtained in short intervals lasting 5 ms and an overlap of 50% taken from a PET noise or OLnoise sample, each of 4 s duration, respectively. Both noise samples, equalized to have the same total RMS value of 1, showed a standard deviation of the short interval RMS of 0.344 in case of PET noise and 0.295 for OLnoise, and an average CREST factor of 2.053 ± 0.330 for PET noise and 2.212 ± 0.314 for OLnoise, i.e., the two temporal characteristic measurements were comparable.

When the ear canal of the KEMAR was blocked, the PET background noise level was reduced: with OHROPAX^®^ Soft to 78.5 dB SPL (60.1 dB(A)) and with OHROPAX^®^ Silicon Aqua to 78.4 dB SPL (60.0 dB(A)). Masking the ear with OHROPAX® reduced noise levels at higher frequencies but had almost no effect on lower and medium frequencies (Fig. 3). Across frequencies, the blocking of the ear canal reduced the level of PET background noise by 14.0 dB(A) (OHROPAX^®^ Soft) and by 14.1 dB(A) (OHROPAX^®^ Aqua Silicon).

We repeated the measurement once, and the mean noise levels varied only marginally with the Nor140: 75.9 dB SPL (65.9 dB(A)). The measured total noise levels differed between the first and second measurements by 1.0 dB SPL for the Nor140, by 0.4 dB SPL for the left ear of the KEMAR (ear canal opened), and by 2.8 dB SPL for the right ear of the KEMAR (ear canal opened). The difference in noise level between the two measurements varied by a frequency of between 6.9 and 2.0 dB SPL for the Nor140, by  -8.0 and 10.3 dB SPL for the left ear of the KEMAR (ear canal opened), and by -6.1 and 12.3 dB SPL for the right ear of the KEMAR (ear canal opened).

**Figure 4:**
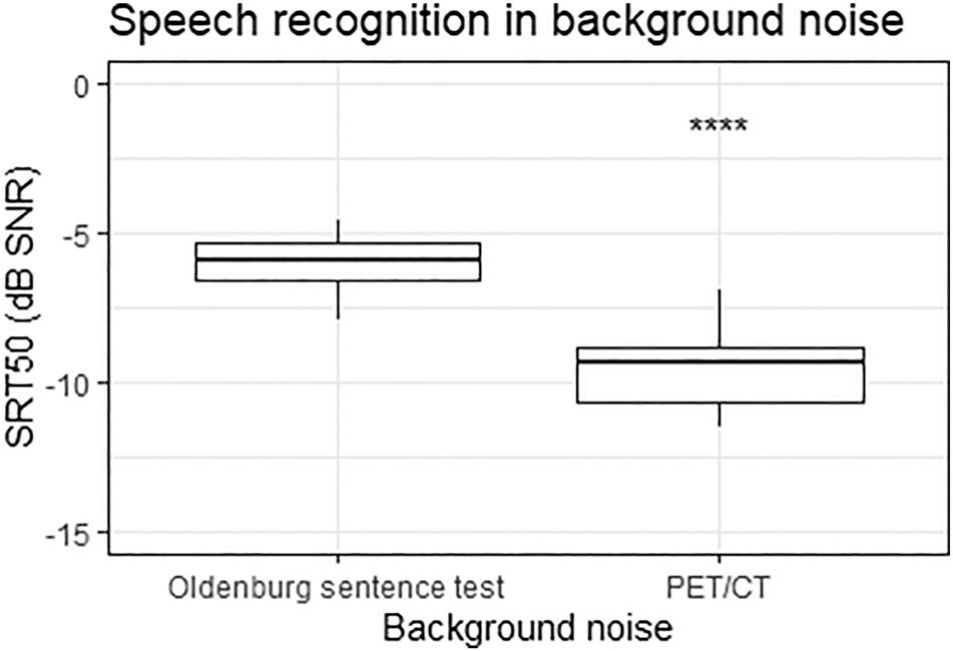
Box-and-whisker plots of the 50% speech reception threshold (SRT50) obtained for sentences in background noise of the Oldenburg sentence test at 76.9 dB SPL or PET noise (Philips Vereos PET/CT) at 76.9 dB SPL in subjects with normal hearing.

Statistical analysis was performed in R. On group average, the subjects with normal hearing demonstrated a speech reception threshold of -6.02 ± 0.97 dB SNR for OLnoise and -9.58 ± 1.36 dB SNR for the PET noise. Individual speech reception thresholds are specified in Table 1. The data were normally distributed according to the Shapiro–Wilk test. A paired t-test revealed a significant lower 50% speech reception threshold for the PET noise compared with the OLnoise (*p* < 0.001). Figure 4 displays box-and-whisker plots of the 50% speech reception threshold.

**Figure 5:**
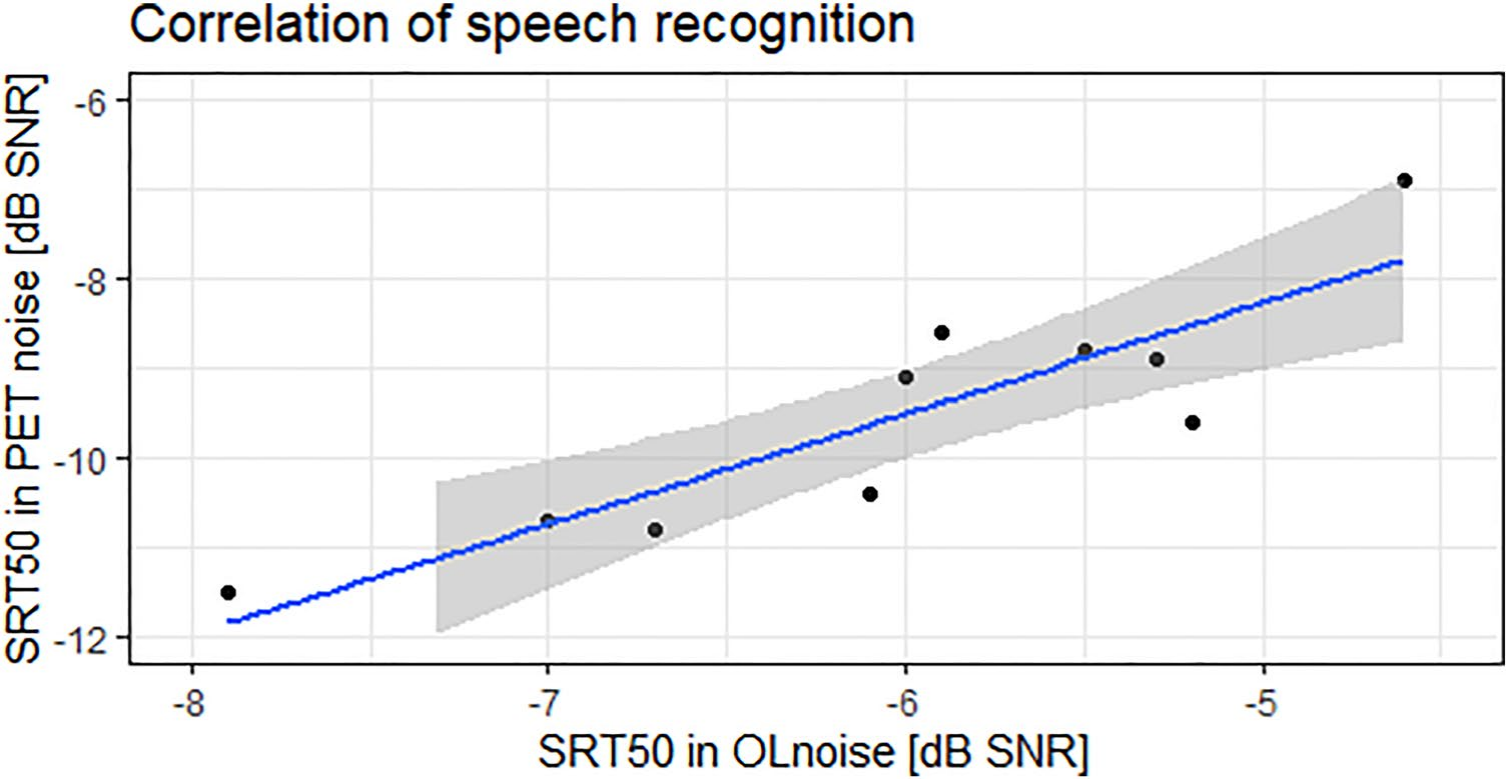
Scatter plot of 50% speech reception threshold (SRT50) obtained for sentences in background noise of the Oldenburg sentence test (OLnoise) or PET noise (Philipps Vereos PET/CT) at 76.9 dB SPL each.

Speech reception thresholds in OLnoise and PET noise correlated significantly according to Spearman (*p* < 0.001, r = 0.88). Figure 5 displays the correlation between speech recognition in OLnoise and PET noise.

## Discussion

In the present study, the PET background noise of air-cooled PET/CT scanners is described for the first time, and the influence of this background noise on speech recognition in subjects with normal hearing is shown.

As PET is used for the investigation of the central neural function of the auditory pathway, background noise is a relevant point for consideration. This is especially true as the background noise level of novel air-cooled PET/CT scanners is considerably higher than that of water-cooled stand-alone PET scanners. In the present study, the noise level was 76.9 dB SPL for the Vereos PET/CT and 75.1 dB SPL (64.5 dB(A)) for the Gemini TF64.

In the retrospective study of Speck et al.^6^, the considerably lower ambient noise of the PET/CT ward (outside the scanner room) was sufficient for auditory stimulation to reveal asymmetric regional glucose metabolism of the inferior colliculi and primary cortices in subjects.

The level of PET background noise is highest in the lower frequencies, and therefore, the masking of the ears with an in-ear solution such as the OHROPAX^®^ enables only a marginal reduction of the overall level. This reduction is mainly present at the higher frequencies. Studies concerning neuronal activity in the central auditory pathway often compare subjects with hearing impairment and subjects with normal hearing. The control of acoustic input in these studies could be crucial. Notably, even with blocked ear canals, a considerable noise level is present resulting in neural activity of the central auditory pathway. This noise-induced activity potentially interferes with the study aim. Hence, a PET scan “in silence” without auditory stimulation is not possible. In a control group with normal hearing, an absence of auditory stimulation in, for example, one ear therefore cannot be achieved with an in-ear solution. Importantly, because low-levels of background noise cannot be achieved when PET/CT scanners are used, the labeling of a condition as “silence” is misleading^12^.

The background noise level of the PET/CT scanner also needs to be considered when presenting auditory stimuli during PET scanning. Background noise results in the environmental degradation of speech because of energetic masking^24^: the target signal (sentences) and nontarget signal (background noise) physically overlap^25^. In energetically constant distractors (both of the noises are compared in the present study), temporal glimpses are rare, leading to the further degradation of speech recognition.

Background noise causes a deterioration of speech recognition in subjects with normal hearing, an effect that is even more pronounced in subjects with a hearing impairment, either bilateral and/or unilateral^20,26,27^. To investigate the specific influence of the background noise from air-cooled PET/CT scanners, speech recognition in noise was tested in young subjects with normal hearing and was compared with a standard test in clinical routine.

The standard test for the assessment of the effect of noise on speech recognition in the German language is the Oldenburg sentence test. The 50% level of speech recognition is the standard value used in otology to measure and describe speech recognition in background noise. The sentences of the Oldenburg sentence test are designed to be grammatically correct but semantically unpredictable and therefore can be repeated several times without being memorized over longer periods of time^19,20^. The OLnoise optimally masks the speech perception of the speech material because of its correspondence to the long-term spectrum of these sentences. The long-term spectrum of the PET noise of the Vereos system is mostly composed of lower frequencies and therefore does not correspond to the long-term spectrum of the Oldenburg sentences.

In the present study, subjects with normal hearing showed decreased speech recognition when exposed to both types of background noise. Knowledge of the amount to which the speech reception threshold in PET noise is lowered will aid the choice of the speech level used for auditory stimuli in the PET/CT scanner, e.g., in order to obtain a speech recognition score of “50% correct”. This needs to be determined beforehand because it is impossible to repeat the auditory stimuli to determine speech recognition during PET scans with auditory stimulation. The reasons for this are: (1) the repetition of heard sentences includes additional brain regions involved with speech production and therefore alters the results derived from auditory stimulation, (2) answers from the subject can lead to changes in head position and result in blurred PET scans, (3) the accuracy of repeated speech cannot be evaluated because of the background noise and physical distance between investigator and subjects (for the radiological protection of the investigator).

Speech recognition in PET background noise produced by the Vereos system was significantly better than in OLnoise. Investigators should therefore consider the differences in speech performance when comparing central auditory activation and speech reception thresholds. As speech recognition in PET noise is better than that in OLnoise, auditory activation might differ. When investigating, for example, subjects with cochlear implants by using PET scanning, the performance and possible classification in good and poor performers should be based on speech recognition in PET noise in order to correlate the central auditory stimulation measured. We have shown that the speech recognition in PET noise is strongly correlated with speech recognition in OLnoise. Therefore, if an assessment of speech performance in PET noise is for some reason not possible or feasible, this performance can be predicted by speech performance obtained in OLnoise. The 50% speech reception threshold in PET noise is 3.5 ± 0.7 dB lower compared with the speech reception threshold in OLnoise. Consequently, a correlation between speech recognition before PET scanning with voxel analysis obtained during PET scanning is presumably rarely associated with a large bias^12^.

The differences in speech reception thresholds can be explained by the smaller masking effect of the PET noise compared with the larger masking effect of the speech-shaped OLnoise.

Signal separation is necessary for speech to be understood when an energetic constant distractor is present. Possible cues that are used to separate signals are (1) common onset, (2) spectral contrast, and (3) harmonicity cues^24^. Both background noises were presented continuously to mirror the situation in the PET/CT scanner; the included subjects were therefore unable to use common onset to separate target and nontarget signals. The spectral contrast between the presented sentences and background noise was greater for PET noise than for the speech-shaped OLnoise. This could have helped speech recognition and explains the better speech reception thresholds in PET noise. Harmonicity cues are present in speech and speech-like noise, like the OLnoise. The absence of harmonicity cues in the PET noise promotes signal separation, and consequently, speech recognition in PET noise was better.

Background noise influences the number of correctly recognized words and, additionally, the reaction time and perceived hearing effort^28^. In the present study, we only measured the speech reception threshold for 50% correct words. In future, an investigation of interest would be to determine the differences in reaction times and hearing effort for speech recognition in OLnoise and PET noise.

## Conclusion

State-of-the-art PET/CT scanners such as the Gemini TF64 and Vereos PET/CT are air-cooled and therefore produce intense noise: 75.1 dB SPL (64.5 dB(A)) for the Gemini TF64 and 76.9 dB SPL (68.4 dB(A)) for the Vereos PET/CT. The PET background noise is mostly composed of low frequencies and can only be marginal reduced by ear plugs. Speech recognition in PET noise is reduced compared with speech recognition in quiet conditions. Nevertheless, speech recognition in PET noise is better than in speech-shaped noise typically applied in clinical routine because of the minor masking effect of the background noise of the PET/CT. The background noise produced by air-cooled PET/CT scanners has to be considered when PET is used for the investigation of the central auditory pathway during speech recognition in quiet or in noise of another type.

## Supplementary Information

Supplementary Information.

## Abbreviations

CBF: Cerebral blood flow
CT: Computed tomography
FDG: Fluorodeoxyglucose
fMRI: Functional magnetic resonance imaging
PET: Positron emission tomography
